# Incidence of emergency neurosurgical TBI procedures: a population-based study

**DOI:** 10.1186/s12873-021-00561-w

**Published:** 2022-01-06

**Authors:** Cathrine Tverdal, Mads Aarhus, Pål Rønning, Ola Skaansar, Karoline Skogen, Nada Andelic, Eirik Helseth

**Affiliations:** 1 Department of Neurosurgery, Oslo University Hospital, P. O. Box 4956 Nydalen, 0424 Oslo, Norway; 2 Faculty of Medicine, Institute of Clinical Medicine, University of Oslo, Boks 1072 Blindern, 0316 Oslo, Norway; 3 Department of Neuroradiology, Oslo University Hospital, P. O. Box 4956 Nydalen, 0424 Oslo, Norway; 4 Department of Physical Medicine and Rehabilitation, Oslo University Hospital, P. O. Box 4956 Nydalen, 0424 Oslo, Norway; 5 Research Centre for Habilitation and Rehabilitation Models and Services (CHARM), Faculty of Medicine, Institute of Health and Society, University of Oslo, Boks 1072 Blindern, 0316 Oslo, Norway

**Keywords:** Emergency, Neurosurgery, Traumatic brain injury, Trauma center, Incidence

## Abstract

**Background:**

The rates of emergency neurosurgery in traumatic brain injury (TBI) patients vary between populations and trauma centers. In planning acute TBI treatment, knowledge about rates and incidence of emergency neurosurgery at the population level is of importance for organization and planning of specialized health care services. This study aimed to present incidence rates and patient characteristics for the most common TBI-related emergency neurosurgical procedures.

**Methods:**

Oslo University Hospital is the only trauma center with neurosurgical services in Southeast Norway, which has a population of 3 million. We extracted prospectively collected registry data from the Oslo TBI Registry – Neurosurgery over a five-year period (2015–2019). Incidence was calculated in person-pears (crude) and age-adjusted for standard population. We conducted multivariate multivariable logistic regression models to assess variables associated with emergency neurosurgical procedures.

**Results:**

A total of 2151 patients with pathological head CT scans were included. One or more emergency neurosurgical procedure was performed in 27% of patients. The crude incidence was 3.9/100,000 person-years. The age-adjusted incidences in the standard population for Europe and the world were 4.0/100,000 and 3.3/100,000, respectively. The most frequent emergency neurosurgical procedure was the insertion of an intracranial pressure monitor, followed by evacuation of the mass lesion. Male sex, road traffic accidents, severe injury (low Glasgow coma score) and CT characteristics such as midline shift and compressed/absent basal cisterns were significantly associated with an increased probability of emergency neurosurgery, while older age was associated with a decreased probability.

**Conclusions:**

The incidence of emergency neurosurgery in the general population is low and reflects neurosurgery procedures performed in patients with severe injuries. Hence, emergency neurosurgery for TBIs should be centralized to major trauma centers.

## Background

The incidence of hospital-admitted patients with traumatic brain injury (TBI) in Western countries is in the range of 83–287 per 100,000 [1–8]. Most commonly, TBI is divided into mild, moderate and severe; mild TBI encompasses the vast majority of cases and can often be cared for at emergency departments or local hospitals. In TBI patients where neurosurgery is indicated, established care pathways to transfer the patients to a level 1 trauma center are utilized. Even though surgery might not be indicated, patients with severe and moderate TBIs tend to benefit from management at centers with expertise and access to neurosurgery and neurosurgical intensive care units [9–11]. Guidelines have been developed to standardize the treatment of TBI, e.g., the Brain Trauma Foundation (BTF) which has published recommendations for the management of severe TBI, including indications for neurosurgical procedures [12, 13]. However, several studies describe a varying degree of compliance with BTF recommendations and neurosurgical management [14–17]. These variations can be explained by differences in the organization of health care, population composition, injury landscape, and available health care resources.

The reported frequencies of hospitalized TBI patients requiring emergency neurosurgery vary between 4 and 29% [18–23]. Such rates are most often based on patients in a study center, and thus they are not representative of the general population, per se. The population-based incidence of emergency neurosurgery for patients with TBI has not yet been reported. Estimation of the incidence of emergency neurosurgery in a defined general population is important to develop adequate care pathways that can be used to predict capacity development, understand resource use and identify high-risk groups for emergency neurosurgery. Such knowledge can assist in the future management of TBI and the distribution of neurosurgical resources, as well as facilitating quality control studies of neurosurgical services, both locally and between countries.

In this study, we present contemporary incidence, rates and patient characteristics for the most common TBI-related emergency neurosurgical procedures in Southeast Norway, encompassing 55% of the Norwegian population. Additionally, we explored the association between demographics, injury-related characteristics and emergency neurosurgical procedures.

## Methods

### Setting and patient population

Oslo University Hospital (OUH) is a Level 1 trauma center and the only hospital with a neurosurgical department in the southeastern region of Norway, which has ≈ 3.0 million inhabitants and covers 110,000 km^2^ with urban and rural areas (current population in Norway is ≈ 5.4 million). The region encompasses 19 local hospitals that provide acute care and general surgical assessment, management and stabilization. Trauma patients with severe injuries or suspected severe TBI are directly transported and admitted to OUH. OUH also serves as the primary trauma referral hospital for Oslo residents (population ≈ 700,000) and manages the Oslo Emergency Department (separate location from the main hospital). All emergency neurosurgical TBI procedures in the southeastern region of Norway are solely performed at OUH (intracranial pressure monitoring, evacuation traumatic intracranial mass lesion, cerebrospinal fluid (CSF) diversion and decompressive craniectomy). Norway provides universal healthcare to all Norwegian residents.

The Oslo TBI Registry – Neurosurgery is a prospective quality control database that has been maintained by the neurosurgical department at OUH since 2015. Data were retrieved manually from electronic medical records and stored in a Medinsight database. To be included in the Oslo TBI Registry – Neurosurgery, all of the following criteria must be fulfilled: (i) traumatic brain injury; (ii) cerebral CT/CTA or cerebral MRI/MRA with findings of acute trauma (hemorrhage, fracture, traumatic axonal injury, vascular injury); (iii) admission to OUH within seven days of injury; and (iv) a Norwegian social security number. A more thorough description of the database and patient characteristics has been previously described [24]. Data were retrieved for patients admitted between January 1, 2015, and December 31, 2019, on September 4, 2020. Population data for the same period were retrieved from the *StatBank* of Statistics Norway [25].

### Variables

The preinjury American Society of Anesthesiologists Physical Status Classification System score (ASA) [26] was grouped into two categories: ASA 1–2 or ASA 3–4. Trauma mechanisms were grouped into (i) falls; (ii) road traffic accidents (RTAs) (including all accidents involving motor vehicles, cyclists and pedestrians); and (iii) others (including assaults, sports, and self-harm). High energy included falls from a height ≥ 3 m, RTAs, or other high-energy accidents. Extracranial injury was registered if there were any simultaneous injuries to other parts of the body, verified by imaging (skeletal fractures or injury to internal organs, not skin injuries) and registered with “no”, “yes, conservative treatment” or “yes, surgical treatment”.

Referrals to OUH were categorized as (i) primary: directly from the scene of an accident; (ii) secondary: initial assessment at a local hospital; and (iii) other: Oslo Emergency Department or other. Trauma team activation: The OUH trauma team is a specially trained interdisciplinary team that systematically assesses the patients upon arrival according to the advanced trauma life support (ATLS) principles [27]. Intubation was registered when performed at the scene of accident, at a local hospital or at admission to OUH. Admission to the intensive care unit (ICU) included all patients admitted to the ICU, whereas uncomplicated short stays (< 24 h) for TBI observation in the intermediate/step-down unit were registered as ward admissions.

The Glasgow coma score (GCS) was recorded as the lowest score documented in the time frame between injury and intubation or arrival at OUH. We grouped patients based on GCS score into mild (13–15), moderate (9–12), and severe (3–8) head injury. Preoperative head CT was assessed and classified based on the Rotterdam CT score [28]. The Rotterdam CT score emphasizes the status of basal cisterns (dichotomized into normal or compressed/absent), midline shift (0–5 mm or > 5 mm), epidural hematoma (present or absent), and traumatic subarachnoid hemorrhage/intraventricular hemorrhage (tSAH/IVH) (present or absent).

We defined “emergency neurosurgical procedure” as undergoing one or more neurosurgical procedures aiming to monitor and/or reduce intracranial pressure (ICP); including insertion of parenchymal ICP monitoring, craniotomy with removal of mass lesions (acute subdural hematoma, epidural hematoma, intracerebral contusion), decompressive hemicraniectomy (DC), or CSF diversion by insertion of external ventricular drains (EVDs). Treatment of TBI at OUH follows the Brain Trauma Foundation guidelines, and indications for surgery are based on international recommendations [13, 29, 30] and presented in Table 1.

**Table 1:** OUH treatment protocol - indication for emergency neurosurgical procedures [29]

Procedure	Indication
ICP-monitor	GCS < 9 and abnormal CT GCS < 9, normal CT and ≥ 2 of following features: age > 40 years or systolic BP < 90 mmHg or GCS Motor (GCS M) < 4 (best side) GCS < 13 and: prolonged surgery in other organ systems expected prolonged ventilator therapy due to other injuries
Evacuation of acute subdural hematoma	GCS < 14 and: hematoma volume > 30 ml or midline shift > 5 mm or hematoma width > 10 mm
Evacuation of epidural hematoma	GCS < 14 and: hematoma volume > 30 ml or midline shift > 5 mm or hematoma width > 15 mm
Evacuation of cerebral contusion	*GCS < 12 and:* contusion volume > 20 ml or midline shift > 5 mm *In case of contusion in the eloquent cortex (motor cortex, language area), decompressive craniectomy should be considered instead of evacuation of the contusion*
CSF diversion	ICP > 22 mmHg for 10 min
	ICP > 25 mmHg for 5 min
Decompressive craniectomy	Persisting ICP > 22 mmHg despite all neuroprotective efforts (*circulation, ventilation, sedation, positioning, temperature regulation, osmotherapy, CSF-diversion*) If evacuation of mass lesion alone does not provide ICP control When CT and clinical presentation are compatible with a meaningful life Age < 60 years

*OUH* Oslo University Hospital, *ICP* intracranial pressure, *GCS* Glasgow coma score, *GCS M* GCS motor score, *CSF* cerebrospinal fluid

### Statistical analysis

Descriptive statistics were used to summarize patient characteristics and emergency neurosurgical procedures. Continuous variables are presented as the mean and standard deviation (SD) or median and percentile, depending on the data distribution. For comparisons between groups, we used the Pearson χ2 test for categorical variables and the independent t-test for continuous variables. Incidence per 100,000 was calculated in person years. For age-adjusted incidence according to the direct method, we used the 2013 European standard population (ESP) and the 2000–2025 WHO World standard population. We conducted multivariable logistic regression models to assess variables associated with emergency neurosurgical procedures. For categorical variables, the category with lowest severity was used as the reference, “no/absent” was coded as 0 and “yes/present” was coded as 1. The results are presented as odds ratios (ORs) with 95% confidence intervals (CIs) and *p*-values. All tests were two-sided, and p-values equal or lower than .05 were considered significant. Data were analyzed using IBM SPSS Statistics, version 25.0 (Armonk, NY: IBM Corp).

### Ethics

The OUH data protection officer (DPO) approved the Medinsight database (approval number 2016/17569) and approved this study as a quality control study (approval number 18/20658).

## Results

Included in this study were 2151 patients with TBI admitted at OUH during the five-year period 2015–2019. The mean age was 52 years (SD 25); 37% were ≥ 65 years, and 68% were males. Preinjury comorbidity with ASA scores ≥3 was registered in 28%. Trauma mechanisms were falls in 1186 (55%) patients, RTA in 488 (23%) patients and other in 477 (22%) patients. The head injury was blunt in 98% and penetrating in 2%. The median time from injury to OUH admittance was 3 hours (IQR 1.3–6.6), and 37% were admitted directly from the scene of the accident. Trauma team triage was performed for the majority of patients (77%), 34% were intubated before arrival or in the ER, and close to half had multiple injuries (47%). TBI was classified as mild, moderate and severe in 59, 15 and 26%, respectively. Patient characteristics are provided in Table 2.

**Table 2:** Demographic and clinical characteristics of the study population

	Overall	Neurosurgery^a^	No-neurosurgery	*p*-value
Total, N (%)	2151 (100)	569 (100)	1582 (100)	
Age, years (mean, SD)	52 (25)	49 (22)	54 (25)	<.001
Male	1466 (68)	425 (75)	1041 (66)	<.001
Preinjury ASA score				
ASA 1–2	1543 (72)	416 (73)	1127 (71)	.39
ASA 3–4	608 (28)	153 (27)	455 (29)	
Antithrombotic therapy	545 (25)	115 (20)	430 (27)	.001
Preinjury substance dependence	325 (15)	113 (20)	212 (13)	<.001
High-energy trauma	810 (38)	281 (49)	529 (33)	<.001
Extracranial injury	1014 (47)	322 (57)	692 (44)	<.001
Alcohol at time of injury	580 (27)	171 (30)	409 (26)	.053
Glasgow coma score (GCS)				
13–15	1267 (59)	108 (19)	1159 (73)	<.001
9–12	334 (15)	111 (20)	223 (14)	
3–8	550 (26)	350 (62)	200 (13)	
CT findings				
Midline shift > 5 mm	353 (16)	216 (38)	137 (9)	<.001
Basal cisterns compressed or absent	350 (16)	228 (40)	122 (8)	<.001
tSAH or IVH	1287 (60)	402 (71)	886 (56)	<.001
EDH	333 (16)	141 (25)	192 (12)	<.001
ASDH	1185 (55)	407 (72)	778 (49)	<.001
Intracerebral contusion	1030 (48)	352 (62)	678 (43)	<.001
CT Rotterdam score				
1–2	848 (39)	133 (23)	715 (45)	<.001
3–4	1122 (52)	335 (59)	787 (50)	
5–6	181 (8)	101 (18)	80 (5)	
Referral to OUH				
Primary	800 (37)	272 (48)	528 (33)	<.001
Secondary	815 (38)	269 (47)	546 (35)	
Other	536 (25)	28 (5)	508 (32)	
Trauma team activation	1655 (77)	533 (94)	1122 (71)	<.001
Intubated	721 (34)	462 (81)	259 (16)	<.001
Any extracranial surgery	459 (21)	171 (30)	288 (18)	<.001
Admitted intensive care unit	1457 (68)	568 (100)	889 (56)	<.001
In-hospital mortality	173 (8)	63 (11)	110 (7)	.002
GCS 15 at discharge	1356 (63)	170 (30)	1186 (75)	<.001

*ASDH* acute subdural hematoma, *EDH* epidural hematoma, *IVH* Intraventricular hemorrhage, *OUH* Oslo University Hospital, *tSAH* Traumatic subarachnoid hemorrhage

Categorical variables presented as N (%), continuous variables presented as the mean (SD) or median (IQR)

^a^Patient undergoing one or more of the following procedures (redo surgeries are not included): ICP-monitor, evacuation of intracranial mass lesion (epidural hematoma, acute subdural hematoma, intracerebral contusion), decompressive craniectomy, external ventricular drain

For further analysis, patients were divided into two groups: the “neurosurgery group” (*N* = 569) who underwent one or more emergency neurosurgical procedures and “no-neurosurgery group” (*N* = 1582) (Table 2). Comparing the two, the neurosurgery group was characterized by younger age, a higher proportion of males, a lower proportion on antithrombotic medication, more high-energy trauma, more extracranial injuries, shorter median time from injury to admittance at OUH, lower GCS score, and higher Rotterdam CT-score. The two groups did not differ with respect to the preinjury ASA score or alcohol influence at the time of injury. The neurosurgery group had higher in-hospital mortality (11% vs 7%, *p* = .002) and had significantly fewer patients with GCS 15 at discharge (30% vs 75%, *p* = <.001).

### Incidence and type of emergency neurosurgical procedures

**Fig. 1:**
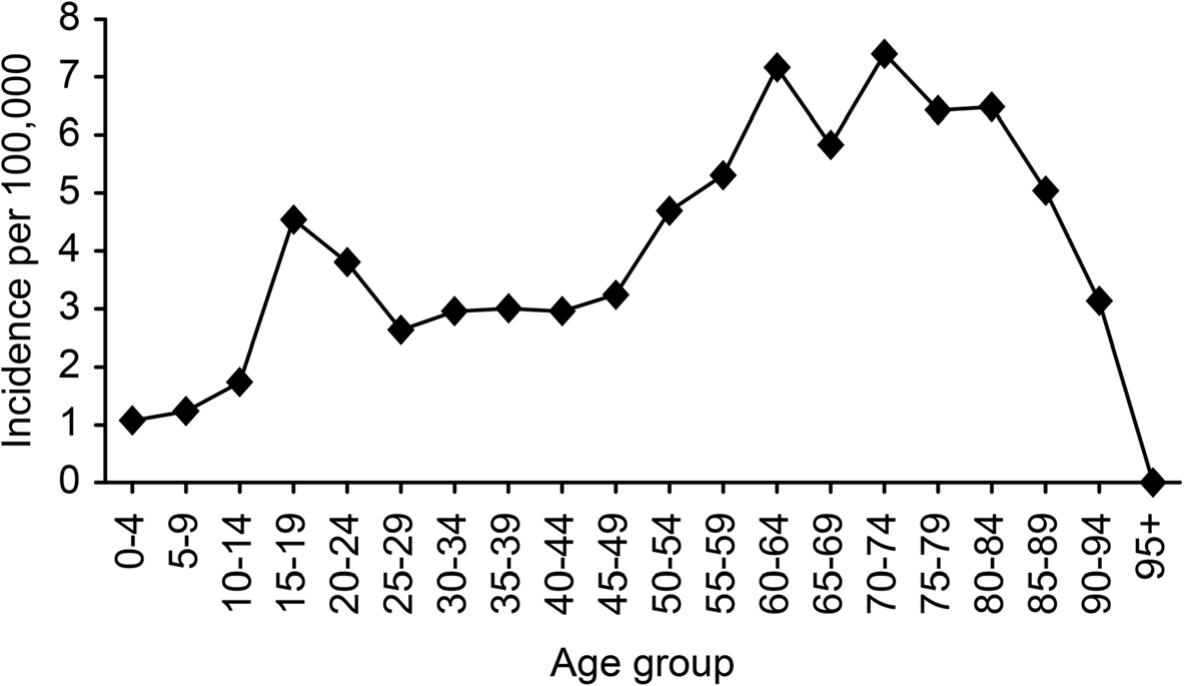
Age-stratified incidence of emergency neurosurgery in the population of Southeast Norway. Incidence was calculated in person years for all age groups, with data from five years (2015–2019). Emergency neurosurgery includes patients undergoing one or more of the following procedures: ICP monitoring, evacuation of intracranial mass lesions, decompressive craniectomy, and external ventricular drain

**Fig. 2:**
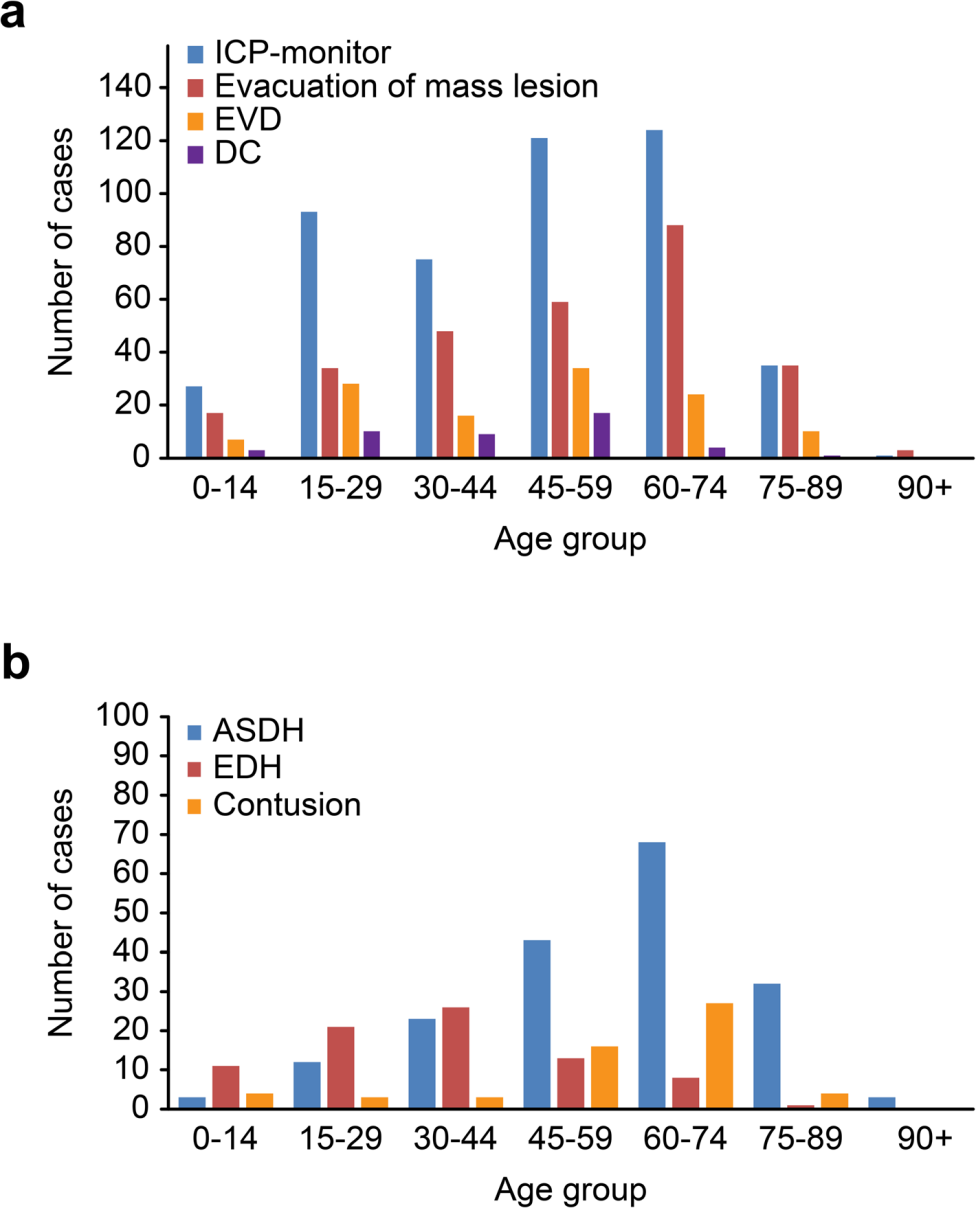
**A** Emergency neurosurgical procedures within age groups. Number of cases by age group undergoing emergency neurosurgery over five years (2015–2019). **B** The type of intracranial mass lesions evacuated within age groups. Redo surgery is not included. Abbreviations ICP: intracranial pressure; EVD: external ventricular drain; DC: decompressive craniectomy; ASDH: acute subdural hematoma; EDH: epidural hematoma

The crude incidence of patients receiving one or more emergency neurosurgical procedures was 3.9/100,000 person-years (Table 3). The age-adjusted incidences in the standard population for Europe and the world were 4.0/100,000 and 3.3/100,000, respectively. Emergency neurosurgical procedures were performed in 27% of patients; this rate varied from 21% (in 2019) to 32.5% (in 2016), and the yearly incidence was in the range of 3.3–4.8/100,000 person-years. Age-specific incidence is presented in Fig. 1, showing a peak incidence between 61 and 84 years. The highest incidence was 7.4/100,000 person-years in the 70–74-year age group. Table 3 and Figs. 2A-B present the incidences and rates for the different neurosurgical procedures.

**Table 3:** Emergency neurosurgical procedures: number of patients, age, male proportion and incidence

	Total, N (%)	Age, mean (SD)	Male, N (%)	Incidence per 100,000, person-years		
				Crude incidence^a^	Age adjusted, Europe^b^	Age adjusted, World^c^
Any procedure	569 (100)	49 (22)	425 (75)	3.87	4.00	3.33
ICP monitor	476 (84)	47 (21)	356 (75)	3.24	3.32	2.84
Evacuation of mass lesion	284 (50)	52 (21)	215 (75)	1.93	2.03	1.55
ASDH	184 (32)	59 (18)	133 (72)	1.25	1.35	0.88
EDH	80 (14)	35 (19)	67 (83)	0.54	0.53	0.58
Intracerebral contusion	57 (10)	56 (18)	42 (74)	0.39	0.41	0.28
EVD	119 (21)	46 (20)	84 (71)	0.81	0.82	0.72
DC	44 (8)	40 (18)	35 (80)	0.30	0.30	0.29

*ASDH* acute subdural hematoma, *EDH* epidural hematoma, *EVD* external ventricular drain, *DC* decompressive craniectomy

Redo surgeries are not included

^a^Population of South-East Norway in person years (2015–2019): 14,740,114

^b^European standard population 2013, EU-27 + EFTA

^c^WHO World standard population: WHO 2000–2025

The most frequent emergency neurosurgical procedure was insertion of an ICP-monitor, done in 476/2151 of admitted patients, and with a population incidence of 3.2/100,000 per person-years (Table 3). The rate of ICP-monitor insertion increased with increasing age, peaked in the 60-year age group and dropped in patients > 75 years (Fig. 2A). This was the only emergency neurosurgical procedure in 39% of cases (184/476). An ICP monitor was inserted in 61% of patients with a GCS ≤8 (336/550) and in 26% of patients with a GCS 9–12 (87/334).

The second most frequent emergency neurosurgical procedure was evacuation of an intracranial mass lesion, performed in 13% (284/2151 patients), resulting in an incidence of 1.9/100,000 person-years (Table 3). The most frequent intracranial mass lesion evacuated was ASDH, followed by EDH and brain contusions (Fig. 2B). The age profile for evacuation of an intracranial mass lesion was similar to that of ICP monitor insertion (Fig. 2A). After stratifying by the three types of intracranial mass lesions evacuated, the age profile differed (Fig. 2B). Evacuation of ASDH was most frequent in patients aged 60–74 years, while evacuation of EDH was most frequent in patients aged < 45 years. Cerebral contusions were most often evacuated in patients aged 45–74 years and rarely in very young or very old patients. In patients with ASDH present on primary CT, the hematomas were evacuated in 16% (184/1185); the corresponding proportions for EDH and intracerebral contusion were 24% (80/333) and 6% (57/1030), respectively. EVD insertion was done in 119/2151 (5.5%) patients resulting in an incidence of 0.8/100,000 person-years, and was rare in all age groups (Table 3 and Fig. 2A). Decompressive craniectomy (DC) was also rarely performed, done in 44/2151 patients (2%), and was mostly performed in patients aged 15–59 years (Table 3 and Fig. 2A). DC was never performed in patients > 80 years.

The multivariable logistic regressions of factors potentially associated with emergency neurosurgery showed that male sex, RTA, low GCS and CT characteristics with midline shift and compressed/absent basal cisterns were significantly associated with an increased probability of emergency neurosurgery, whereas high age (> 75 years) was associated with decreased probability. These results are presented in Table 4. The pattern was similar for insertion of an ICP-monitor, but for this procedure, the most significant association was for low GCS 3–8 (OR 29.1, 95% CI 20.3, 41.7). For evacuation of intracranial mass lesions, the presence of a midline shift was the strongest factor (OR 19.2, 95% CI 12.9, 28.7).

**Table 4:** Multivariable logistic regression of potential factors associated with emergency neurosurgical procedures, placement of ICP-monitor and evacuation of mass lesion

Variable	Emergency neurosurgery^a^		ICP-monitor		Evacuation mass lesion	
	OR (95% CI)	*p*-value	OR (95% CI)	*p*-value	OR (95% CI)	*p*-value
Age						
0–14 years	1		1		1	
15–29 years	1.15 (.68, 1.96)	.60	1.43 (.79, 2.60)	.24	.62 (.30, 1.28)	.20
30–44 years	1.06 (.61, 1.83)	.84	1.17 (.63, 2.17)	.63	.81 (.40, 1.63)	.55
45–59 years	1.29 (.77, 2.17)	.33	2.10 (1.16. 3.77)	.01	.54 (.27, 1.08)	.08
60–74 years	1.10 (.65, 1.87)	.72	1.44 (.79, 2.62)	.23	.52 (.26, 1.04)	.07
75–89 years	.41 (.22, .77)	.00	.38 (.18, .78)	.01	.23 (.10, .52)	<.001
90+ years	.10 (.02, .43)	.01	.05 (.01, .47)	.01	.14 (.03, .60)	.01
Sex						
Female	1		1		1	
Male	1.49 (1.13, 1.97)	.01	1.48 (1.05, 1.94)	.02	1.86 (1.28, 2.70)	.00
ASA-score						
1–2	1		1		1	
3–4	.92 (.66, 1.29)	.63	.76 (.52, 1.09)	.14	1.33 (.88, 2.01)	.18
Antithrombotic						
None	1		1		1	
Plate inhibitor	1.02 (.64, 1.60)	.95	1.48 (.90, 2.43)	.13	.72 (.39, 1.31)	.28
Anticoagulation	.75 (.43, 1.31)	.32	.88 (.48, 1.62)	.69	.63 (.34, 1.18)	.15
Combination	2.20 (1.06, 4.54)	.03	1.57 (.64, 3.89)	.33	1.57 (.66, 3.71)	.31
Trauma type						
Fall	1		1		1	
RTA	1.76 (1.29, 2.40)	<.001	1.97 (1.40, 2.77)	<.001	1.10 (.71, 1.70)	.67
Other	1.16 (.84, 1.60)	.37	1.15 (.81, 1.63)	.43	.92 (.61, 1.40)	.70
GCS						
13–15	1		1		1	
9–12	4.40 (3.19. 6.05)	<.001	7.17 (4.89, 10.51)	<.001	2.21 (1.44, 3.41)	<.001
3–8	12.47 (9.27, 16.77)	<.001	29.12 (20.34, 41.69)	<.001	1.27 (.83, 1.95)	.27
Midline shift						
No (≤5 mm)	1		1		1	
Yes (> 5 mm)	3.69 (2.58, 5.29)	<.001	1.50 (1.01, 2.23)	.05	19.23 (12.88, 28.70)	<.001
Basal cisterns						
Normal	1		1		1	
Compressed/absent	1.75 (1.23, 2.50)	.00	1.20 (1.36, 2.92)	<.001	2.52 (1.63, 3.88)	<.001

*OR* odds ratio, *CI* confidence interval

^a^Patient undergoing one or more of the following procedures (redo surgeries are not included): ICP-monitor, evacuation of mass lesion, decompressive craniectomy, external ventricular drain

## Discussion

The incidence of emergency neurosurgery for TBI in the general Norwegian population was 3.9/100,000 person years over the five-year study period. Insertion of an ICP-monitor was the most frequent procedure, followed by evacuation of an intracranial mass lesion. Overall, emergency neurosurgery was associated with male sex, RTA, low GCS and CT characteristics with midline shift and compressed/absent basal cisterns. The incidence of emergency neurosurgery decreased in elderly patients.

The frequencies of emergency neurosurgery are in accordance with previous research comparing subgroups within the TBI population [18–23]. However, to our knowledge, this study is the first to describe a general population-based incidence of emergency neurosurgery. We found the incidence of emergency neurosurgery (3.9/100,000 person-years) to be quite low compared to the reported incidence of hospital-admitted TBI patients (83–287/100,000) [1–8]. To identify the few patients in need of neurosurgery, screening with head CT is performed with a rather low threshold, e.g., according to the Scandinavian guidelines for the initial management of minimal, mild and moderate head injuries [31]. Thus, minor lesions less likely to require emergency neurosurgery are frequently identified.

The incidence of emergency neurosurgery varied with age; it was low in children and peaked in the 60–70 year age group. To some extent, this reflects the epidemiological shift described over the last decade in high-income countries – the typical TBI patient has changed from a young male, injured in a high-energy trauma, to an elderly man or woman, often with significant comorbidity, injured in a low-energy fall [21, 23, 32, 33]. Still, male sex was significantly associated with an increased probability of emergency neurosurgery in this study. Males are seemingly more likely to take risks [34], which may explain this overrepresentation. It must also be taken into consideration that the type of injury mechanism differs globally; in low- and middle-income countries, RTA is the most common cause and access to neurosurgery is more limited [10]. The mean age of TBI patients undergoing one or more neurosurgical procedures was significantly lower than the mean age of all admitted TBI patients. This can partly be explained by difference in trauma mechanism, younger patients are more often involved in high-energy trauma (e.g. RTA) [24] and receive ICP-monitoring. The incidence of emergency neurosurgery declined abruptly in patients above 85 years, which was somewhat unexpected since the incidence of TBI-related hospital admissions is highest for the eldest patients [21, 32]. Decisions to limit treatment are more often made for the oldest patients [35–37], and for many of these patients, it is justified to refrain from emergency neurosurgery based on poor prognosis, severe comorbidity, and frailty [38, 39]. However, the use of age alone as a criterion for treatment limitation must be practiced with caution, since several studies have shown that older patients may benefit from aggressive treatment and access to rehabilitation [40–46].

The most frequently performed emergency neurosurgical procedure was the insertion of an ICP monitor. According to the BTF guidelines, ICP monitoring is recommended for all salvageable TBI patients with an abnormal head CT and a GCS ≤8 [12]. In our study, GCS ≤8 was the strongest factor associated with insertion of an ICP monitor; 61% of patients with GCS ≤8 received an ICP monitor. This is in line with a European multicenter study where the proportion was 62% [23]. In North American studies, the rate of ICP monitoring of severe TBI patients ranges from 10 to 65% [15, 16, 47]. In our study, 26% of patients with GCS 9–12 also received an ICP monitor. The indication for ICP monitoring in this group was mainly prolonged surgery or expected prolonged ventilator treatment due to other injuries, which is in accordance with our local protocol.

Evacuation of intracranial mass lesions was the second most frequent emergency neurosurgical procedure, with an incidence of 1.9/100,000. Overall, evacuation of mass lesions was performed in 13% of CT-verified TBI patients admitted to our institution, which is in line with other studies with similar patient populations, ranging from 9 to 18% [19, 21, 23]. ASDH was the most frequent mass lesion evacuated; it was performed in all age groups but more often in elderly patients, which is in line with other studies [23, 32, 48, 49]. In our study, 16% of the patients with ASDH present on primary CT underwent craniotomy with evacuation of ASDH, which is in line with the previous reports [18]. EDH is known to be more frequent in younger people because the dura adheres more tightly to the skull with age [30]. Thus, as expected, evacuation of EDH was most often performed in patients aged < 45 years. Of patients with EDH present on CT scan, 24% had a craniotomy, which is somewhat higher than the previously reported rate of 17% [18]. Evacuation of cerebral contusions was most often done in patients aged 45–74 years, rarely in the younger and older patient groups, and in only 6% of those with contusion present on CT scan. This corresponds to the 2–10% reported by others [23, 32, 49]. In the majority of patients observed in the hospital for a traumatic intracranial lesion, the lesion will not progress to a size requiring surgical evacuation. However, the results show that close observation of patients with intracranial mass lesions are necessary. Moreover, given the low incidence of emergency neurosurgery, it is clear that only designated centers can provide competent neurosurgical services.

In line with other studies, low GCS and CT characteristics such as midline shift and compressed basal cisterns, were strong predictors for emergency neurosurgery [13, 48, 49]. A midline shift ≥5 mm on cerebral CT was the predominant factor associated with evacuation of mass lesions, along with compressed basal cisterns. The size of the mass lesions was not measured in our study, but the degree of midline shift and the status of the basal cisterns are good indirect measures of the volume of intracranial mass lesions. At our institution, EVDs are primarily used to reduce elevated ICP and not to monitor ICP. The main reason behind this treatment strategy is the risk of infection associated with EVD, and that an intraparenchymal ICP sensor causes less surgical trauma [50–52]. Decompressive craniectomy (DC) for severe TBI is still regarded as a treatment with limited documented benefit and rarely documented in patients ≥65 years [43, 44, 53–55]. The two main indications for DC at our institution have been as a last resort management option for refractory raised ICP and in cases with severe intraoperative brain swelling, similar to other European trauma centers [17] and guidelines [56, 57].

### Strength and limitations

This study presents a population-based incidence of TBI emergency neurosurgical procedures over a total period of five years from a defined geographical region covering both large rural and urban areas. The region has a defined written criterion for emergency neurosurgery and a stable all-hour presence of neurosurgeons. Data were retrieved manually, thus avoiding bias of potential medical coding errors, which is a risk with aggregated data from national registries.

A limitation of this study is the lack of detailed information about the anatomical localization or volumetry of the traumatic intracranial mass lesions. Moreover, the study is restricted to what we defined as emergency neurosurgical procedures. Hence, we do not describe all neurosurgical procedures relevant for TBI patients; e.g., cranioplasty, dural repair or redo-surgery, and surgery at later stages, such as replacement of bone flaps, shunts and chronic subdural hematoma, were not included.

## Conclusion

The incidence of emergency neurosurgery after TBI in the general population is low. Emergency neurosurgery is multifaceted, but significant factors associated with surgery were male sex, road traffic accidents, and severe TBI. The low incidence must be taken in consideration when organizing trauma care and neurosurgical services. To maintain necessary expertise, emergency neurosurgery should be centralized to major trauma center with adequate resources, staffing levels and neurosurgery training.

## Data Availability

The datasets generated and/or analyzed during the current study are not publicly available due to the sensitivity of the material, but they are available from the corresponding author on reasonable request.

## Abbreviations

ASA-PS: American Society of Anesthesiologists Physical Status Classification System score
ASDH: Acute subdural hematoma
CI: Confidence interval
CCP: Cerebral perfusion pressure
CSF: Cerebrospinal fluid
CT: Computed tomography
DC: Decompressive craniectomy
DPO: Data protection officer
ED: Emergency department
EDH: Epidural hematoma
EVD: External ventricular drainage
GCS: Glasgow coma score
ICP: Intracranial pressure
ICU: Intensive care unit
IVH: Intraventricular hemorrhage
LOS: Length of stay
RTA: Road traffic accident
OR: Odds ratio
OUH: Oslo University Hospital
TBI: Traumatic brain injury
tSAH: Traumatic subarachnoid hemorrhage

## References

[1] Peeters W, van den Brande R, Polinder S, Brazinova A, Steyerberg EW, Lingsma HF, Maas AI (2015). Epidemiology of traumatic brain injury in Europe. Acta Neurochir.

[2] Centers for Disease Control and Prevention (2019). Surveillance Report of Traumatic Brain Injury-related Emergency Department Visits, Hospitalizations, and Deaths—United States, 2014. Centers for Disease Control and Prevention.

[3] Koskinen S, Alaranta H (2008). Traumatic brain injury in Finland 1991-2005: a nationwide register study of hospitalized and fatal TBI. Brain Inj.

[4] Pedersen K, Fahlstedt M, Jacobsson A, Kleiven S, von Holst H (2015). A National Survey of traumatic brain injuries admitted to hospitals in Sweden from 1987 to 2010. Neuroepidemiology.

[5] Andelic N, Sigurdardottir S, Brunborg C, Roe C (2008). Incidence of hospital-treated traumatic brain injury in the Oslo population. Neuroepidemiology.

[6] Heskestad B, Baardsen R, Helseth E, Romner B, Waterloo K, Ingebrigtsen T (2009). Incidence of hospital referred head injuries in Norway: a population based survey from the Stavanger region. Scand J Trauma Resusc Emerg Med.

[7] Rickels E, von Wild K, Wenzlaff P (2010). Head injury in Germany: a population-based prospective study on epidemiology, causes, treatment and outcome of all degrees of head-injury severity in two distinct areas. Brain Inj.

[8] Majdan M, Plancikova D, Brazinova A, Rusnak M, Nieboer D, Feigin V, Maas A (2016). Epidemiology of traumatic brain injuries in Europe: a cross-sectional analysis. Lancet Public Health.

[9] Badjatia N, Carney N, Crocco TJ, Fallat ME, Hennes HM, Jagoda AS, Jernigan S, Letarte PB, Lerner EB, Moriarty TM, Pons PT, Sasser S, Scalea T, Schleien CL, Wright DW, Brain Trauma Foundation, BTF Center for Guidelines Management (2008). Guidelines for prehospital management of traumatic brain injury 2nd edition. Prehosp Emerg Care.

[10] Maas AIR, Menon DK, Adelson PD, Andelic N, Bell MJ, Belli A, Bragge P, Brazinova A, Büki A, Chesnut RM, Citerio G, Coburn M, Cooper DJ, Crowder AT, Czeiter E, Czosnyka M, Diaz-Arrastia R, Dreier JP, Duhaime AC, Ercole A, van Essen TA, Feigin VL, Gao G, Giacino J, Gonzalez-Lara LE, Gruen RL, Gupta D, Hartings JA, Hill S, Jiang JY, Ketharanathan N, Kompanje EJO, Lanyon L, Laureys S, Lecky F, Levin H, Lingsma HF, Maegele M, Majdan M, Manley G, Marsteller J, Mascia L, McFadyen C, Mondello S, Newcombe V, Palotie A, Parizel PM, Peul W, Piercy J, Polinder S, Puybasset L, Rasmussen TE, Rossaint R, Smielewski P, Söderberg J, Stanworth SJ, Stein MB, von Steinbüchel N, Stewart W, Steyerberg EW, Stocchetti N, Synnot A, te Ao B, Tenovuo O, Theadom A, Tibboel D, Videtta W, Wang KKW, Williams WH, Wilson L, Yaffe K, Adams H, Agnoletti V, Allanson J, Amrein K, Andaluz N, Anke A, Antoni A, van As AB, Audibert G, Azaševac A, Azouvi P, Azzolini ML, Baciu C, Badenes R, Barlow KM, Bartels R, Bauerfeind U, Beauchamp M, Beer D, Beer R, Belda FJ, Bellander BM, Bellier R, Benali H, Benard T, Beqiri V, Beretta L, Bernard F, Bertolini G, Bilotta F, Blaabjerg M, den Boogert H, Boutis K, Bouzat P, Brooks B, Brorsson C, Bullinger M, Burns E, Calappi E, Cameron P, Carise E, Castaño-León AM, Causin F, Chevallard G, Chieregato A, Christie B, Cnossen M, Coles J, Collett J, Della Corte F, Craig W, Csato G, Csomos A, Curry N, Dahyot-Fizelier C, Dawes H, DeMatteo C, Depreitere B, Dewey D, van Dijck J, Đilvesi Đ, Dippel D, Dizdarevic K, Donoghue E, Duek O, Dulière GL, Dzeko A, Eapen G, Emery CA, English S, Esser P, Ezer E, Fabricius M, Feng J, Fergusson D, Figaji A, Fleming J, Foks K, Francony G, Freedman S, Freo U, Frisvold SK, Gagnon I, Galanaud D, Gantner D, Giraud B, Glocker B, Golubovic J, Gómez López PA, Gordon WA, Gradisek P, Gravel J, Griesdale D, Grossi F, Haagsma JA, Håberg AK, Haitsma I, van Hecke W, Helbok R, Helseth E, van Heugten C, Hoedemaekers C, Höfer S, Horton L, Hui J, Huijben JA, Hutchinson PJ, Jacobs B, van der Jagt M, Jankowski S, Janssens K, Jelaca B, Jones KM, Kamnitsas K, Kaps R, Karan M, Katila A, Kaukonen KM, de Keyser V, Kivisaari R, Kolias AG, Kolumbán B, Kolundžija K, Kondziella D, Koskinen LO, Kovács N, Kramer A, Kutsogiannis D, Kyprianou T, Lagares A, Lamontagne F, Latini R, Lauzier F, Lazar I, Ledig C, Lefering R, Legrand V, Levi L, Lightfoot R, Lozano A, MacDonald S, Major S, Manara A, Manhes P, Maréchal H, Martino C, Masala A, Masson S, Mattern J, McFadyen B, McMahon C, Meade M, Melegh B, Menovsky T, Moore L, Morgado Correia M, Morganti-Kossmann MC, Muehlan H, Mukherjee P, Murray L, van der Naalt J, Negru A, Nelson D, Nieboer D, Noirhomme Q, Nyirádi J, Oddo M, Okonkwo DO, Oldenbeuving AW, Ortolano F, Osmond M, Payen JF, Perlbarg V, Persona P, Pichon N, Piippo-Karjalainen A, Pili-Floury S, Pirinen M, Ple H, Poca MA, Posti J, van Praag D, Ptito A, Radoi A, Ragauskas A, Raj R, Real RGL, Reed N, Rhodes J, Robertson C, Rocka S, Røe C, Røise O, Roks G, Rosand J, Rosenfeld JV, Rosenlund C, Rosenthal G, Rossi S, Rueckert D, de Ruiter GCW, Sacchi M, Sahakian BJ, Sahuquillo J, Sakowitz O, Salvato G, Sánchez-Porras R, Sándor J, Sangha G, Schäfer N, Schmidt S, Schneider KJ, Schnyer D, Schöhl H, Schoonman GG, Schou RF, Sir Ö, Skandsen T, Smeets D, Sorinola A, Stamatakis E, Stevanovic A, Stevens RD, Sundström N, Taccone FS, Takala R, Tanskanen P, Taylor MS, Telgmann R, Temkin N, Teodorani G, Thomas M, Tolias CM, Trapani T, Turgeon A, Vajkoczy P, Valadka AB, Valeinis E, Vallance S, Vámos Z, Vargiolu A, Vega E, Verheyden J, Vik A, Vilcinis R, Vleggeert-Lankamp C, Vogt L, Volovici V, Voormolen DC, Vulekovic P, Vande Vyvere T, van Waesberghe J, Wessels L, Wildschut E, Williams G, Winkler MKL, Wolf S, Wood G, Xirouchaki N, Younsi A, Zaaroor M, Zelinkova V, Zemek R, Zumbo F (2017). Traumatic brain injury: integrated approaches to improve prevention, clinical care, and research. Lancet Neurol.

[11] Sollid S, Sundstrom T, Ingebrigtsen T, Romner B, Wester K (2009). Organisation of traumatic head injury management in the Nordic countries. Emerg Med J.

[12] Carney N, Totten AM, O'Reilly C, Ullman JS, Hawryluk GW, Bell MJ, Bratton SL, Chesnut R, Harris OA, Kissoon N (2017). Guidelines for the Management of Severe Traumatic Brain Injury, Fourth Edition. Neurosurgery.

[13] Bullock M, Chesnut R, Ghajar J, Gordon D, Hartl R, Newell DW, Servadei F, Walters BC, Wilberger JJN (2006). Surgical management of traumatic brain injury. Neurosurgery.

[14] Cnossen MC, Polinder S, Andriessen TM, van der Naalt J, Haitsma I, Horn J, Franschman G, Vos PE, Steyerberg EW, Lingsma H (2017). Causes and consequences of treatment variation in moderate and severe traumatic brain injury: a multicenter study. Crit Care Med.

[15] Hoffman H, Bunch KM, Furst T, Chin LS (2020). Use of intracranial pressure monitoring in patients with severe traumatic brain injury. World Neurosurg.

[16] Piccinini A, Lewis M, Benjamin E, Aiolfi A, Inaba K, Demetriades D (2017). Intracranial pressure monitoring in severe traumatic brain injuries: a closer look at level 1 trauma centers in the United States. Injury.

[17] van Essen TA, den Boogert HF, Cnossen MC, de Ruiter GCW, Haitsma I, Polinder S, Steyerberg EW, Menon D, Maas AIR, Lingsma HF (2018). Variation in neurosurgical management of traumatic brain injury: a survey in 68 centers participating in the CENTER-TBI study. Acta Neurochir.

[18] Esposito TJ, Reed RL, Gamelli RL, Luchette FA (2005). Neurosurgical coverage: essential, desired, or irrelevant for good patient care and trauma center status. Ann Surg.

[19] Joseph B, Haider AA, Pandit V, Tang A, Kulvatunyou N, O’Keeffe T, Rhee P (2015). Changing paradigms in the management of 2184 patients with traumatic brain injury. Ann Surg.

[20] Lecky FE, Russell W, McClelland G, Pennington E, Fuller G, Goodacre S, Han K, Curran A, Holliman D, Chapman N, Freeman J, Byers S, Mason S, Potter H, Coats T, Mackway-Jones K, Peters M, Shewan J (2017). Bypassing nearest hospital for more distant neuroscience care in head-injured adults with suspected traumatic brain injury: findings of the head injury transportation straight to neurosurgery (HITS-NS) pilot cluster randomised trial. BMJ Open.

[21] Maegele M, Lefering R, Sakowitz O, Kopp MA, Schwab JM, Steudel WI, Unterberg A, Hoffmann R, Uhl E, Marzi I (2019). The incidence and management of moderate to severe head injury. Deutsches Arzteblatt Int.

[22] Stranjalis G, Bouras T, Korfias S, Andrianakis I, Pitaridis M, Tsamandouraki K, Alamanos Y, Sakas DE, Marmarou A (2008). Outcome in 1,000 head injury hospital admissions: the Athens head trauma registry. J Trauma.

[23] Steyerberg EW, Wiegers E, Sewalt C, Buki A, Citerio G, De Keyser V, Ercole A, Kunzmann K, Lanyon L, Lecky F (2019). Case-mix, care pathways, and outcomes in patients with traumatic brain injury in CENTER-TBI: a European prospective, multicentre, longitudinal, cohort study. Lancet Neurol.

[24] Tverdal C, Aarhus M, Andelic N, Skaansar O, Skogen K, Helseth E (2020). Characteristics of traumatic brain injury patients with abnormal neuroimaging in Southeast Norway. Inj Epidemiol.

[25] Statistics Norway. Statbank - Population. Available from:https://www.ssb.no/en/statbank/table/01222Accessed 29 Apr 2020.

[26] American Society of Anesthesiologists. ASA Physical Status Classification System 2014. Available from:https://www.asahq.org/standards-and-guidelines/asa-physical-status-classification-system Accessed 11 Aug 2019.

[27] ATLS (2018). Advanced trauma life support : student course manual. Advanced trauma life support student course manual.

[28] Maas AI, Hukkelhoven CW, Marshall LF, Steyerberg EW (2005). Prediction of outcome in traumatic brain injury with computed tomographic characteristics: a comparison between the computed tomographic classification and combinations of computed tomographic predictors. Neurosurgery.

[29] Aarhus M, Helseth E, Sunde K. Traumemanualen. Hodeskader [Head injuries]: Oslo University Hospital; 2016. Available fromhttps://www.traumemanualen.no/index.php?action=showtopic&topic=JZMwkGD3] Accessed 19 Oct 2020.

[30] Sundstrøm T, Grände PO, Luoto T, Rosenlund C, Undén J, Wester KG (2020). Management of Severe Traumatic Brain Injury.

[31] Unden J, Ingebrigtsen T, Romner B (2013). Scandinavian guidelines for initial management of minimal, mild and moderate head injuries in adults: an evidence and consensus-based update. BMC Med.

[32] Posti JP, Sipilä JOT, Luoto TM, Rautava P, Kytö V (2020). A decade of geriatric traumatic brain injuries in Finland: population-based trends. Age Ageing.

[33] Stocchetti N, Paternò R, Citerio G, Beretta L, Colombo A (2012). Traumatic brain injury in an aging population. J Neurotrauma.

[34] Byrnes JP, Miller DC, Schafer WD (1999). Gender differences in risk taking: a meta-analysis. Psychol Bull.

[35] Jochems D, van Wessem KJP, Houwert RM, Brouwers HB, Dankbaar JW, van Es MA, Geurts M, Slooter AJC, Leenen LPH (2018). Outcome in patients with isolated moderate to severe traumatic brain injury. Crit Care Res Pract.

[36] Robertsen A, Forde R, Skaga NO, Helseth E (2017). Treatment-limiting decisions in patients with severe traumatic brain injury in a Norwegian regional trauma center. Scand J Trauma Resusc Emerg Med.

[37] Skaansar O, Tverdal C, Rønning PA, Skogen K, Brommeland T, Røise O, Aarhus M, Andelic N, Helseth E (2020). Traumatic brain injury-the effects of patient age on treatment intensity and mortality. BMC Neurol.

[38] Castillo-Angeles M, Cooper Z, Jarman MP, Sturgeon D, Salim A, Havens JM (2020). Association of Frailty with Morbidity and Mortality in emergency general surgery by procedural risk level. JAMA Surg.

[39] George EL, Hall DE, Youk A, Chen R, Kashikar A, Trickey AW, Varley PR, Shireman PK, Shinall MC, Massarweh NN (2020). Association between patient frailty and postoperative mortality across multiple noncardiac surgical specialties. JAMA Surg.

[40] Anke A, Andelic N, Skandsen T, Knoph R, Ader T, Manskow U, Sigurdardottir S, Roe C (2015). Functional recovery and life satisfaction in the first year after severe traumatic brain injury: a prospective multicenter study of a Norwegian National Cohort. J Head Trauma Rehabil.

[41] Lilley EJ, Williams KJ, Schneider EB, Hammouda K, Salim A, Haider AH, Cooper Z (2016). Intensity of treatment, end-of-life care, and mortality for older patients with severe traumatic brain injury. J Trauma Acute Care Surg.

[42] Mak CH, Wong SK, Wong GK, Ng S, Wang KK, Lam PK, Poon WS (2012). Traumatic brain injury in the elderly: is it as bad as we think. Curr Transl Geriatr Exp Gerontol Rep.

[43] Roe C, Skandsen T, Manskow U, Ader T, Anke A (2015). Mortality and one-year functional outcome in elderly and very old patients with severe traumatic brain injuries: observed and predicted. Behav Neurol.

[44] Taussky P, Hidalgo ET, Landolt H, Fandino J (2012). Age and salvageability: analysis of outcome of patients older than 65 years undergoing craniotomy for acute traumatic subdural hematoma. World Neurosurg.

[45] Whitmore RG, Thawani JP, Grady MS, Levine JM, Sanborn MR, Stein SC (2012). Is aggressive treatment of traumatic brain injury cost-effective. J Neurosurg.

[46] Younsi A, Fischer J, Habel C, Riemann L, Scherer M, Unterberg A, Zweckberger K (2020). Mortality and functional outcome after surgical evacuation of traumatic acute subdural hematomas in octa- and nonagenarians. Eur J Trauma Emerg Surg.

[47] Dawes AJ, Sacks GD, Cryer HG, Gruen JP, Preston C, Gorospe D, Cohen M, McArthur DL, Russell MM, Maggard-Gibbons M, Ko CY, Los Angeles County Trauma Consortium (2015). Compliance with evidence-based guidelines and Interhospital variation in mortality for patients with severe traumatic brain injury. JAMA Surg.

[48] Gómez PA, Castaño-León AM, Lora D, Cepeda S, Lagares A (2017). Trends in computed tomography characteristics, intracranial pressure monitoring and surgical management in severe traumatic brain injury: analysis of a data base of the past 25 years in a neurosurgery department. Neurocirugia (Astur).

[49] Rossi-Mossuti F, Fisch U, Schoettker P, Gugliotta M, Morard M, Schucht P, Schatlo B, Levivier M, Walder B, Fandino J (2016). Surgical treatment of severe traumatic brain injury in Switzerland: results from a multicenter study. J Neurol Surg Part A Cent Eur Neurosurg.

[50] Hoffman H, Jalal MS, Chin LS (2019). The incidence of meningitis in patients with traumatic brain injury undergoing external ventricular drain placement: a Nationwide inpatient sample analysis. Neurocrit Care.

[51] Beer R, Lackner P, Pfausler B, Schmutzhard E (2008). Nosocomial ventriculitis and meningitis in neurocritical care patients. J Neurol.

[52] Sorinola A, Buki A, Sandor J, Czeiter E (2019). Risk factors of external ventricular drain infection: proposing a model for future studies. Front Neurol.

[53] Cooper DJ, Rosenfeld JV, Murray L, Arabi YM, Davies AR, D'Urso P, Kossmann T, Ponsford J, Seppelt I, Reilly P, Wolfe R (2011). Decompressive craniectomy in diffuse traumatic brain injury. N Engl J Med.

[54] Hutchinson PJ, Kolias AG, Timofeev IS, Corteen EA, Czosnyka M, Timothy J, Anderson I, Bulters DO, Belli A, Eynon CA, Wadley J, Mendelow AD, Mitchell PM, Wilson MH, Critchley G, Sahuquillo J, Unterberg A, Servadei F, Teasdale GM, Pickard JD, Menon DK, Murray GD, Kirkpatrick PJ (2016). Trial of decompressive Craniectomy for traumatic intracranial hypertension. N Engl J Med.

[55] Sahuquillo J, Dennis JA (2019). Decompressive craniectomy for the treatment of high intracranial pressure in closed traumatic brain injury. Cochrane Database Syst Rev.

[56] Hawryluk GWJ, Rubiano AM, Totten AM, O'Reilly C, Ullman JS, Bratton SL, Chesnut R, Harris OA, Kissoon N, Shutter L (2020). Guidelines for the management of severe traumatic brain injury: 2020 update of the decompressive craniectomy recommendations. Neurosurgery.

[57] Hutchinson PJ, Kolias AG, Tajsic T, Adeleye A, Aklilu AT, Apriawan T, Bajamal AH, Barthélemy EJ, Devi BI, Bhat D, Bulters D, Chesnut R, Citerio G, Cooper DJ, Czosnyka M, Edem I, el-Ghandour NMF, Figaji A, Fountas KN, Gallagher C, Hawryluk GWJ, Iaccarino C, Joseph M, Khan T, Laeke T, Levchenko O, Liu B, Liu W, Maas A, Manley GT, Manson P, Mazzeo AT, Menon DK, Michael DB, Muehlschlegel S, Okonkwo DO, Park KB, Rosenfeld JV, Rosseau G, Rubiano AM, Shabani HK, Stocchetti N, Timmons SD, Timofeev I, Uff C, Ullman JS, Valadka A, Waran V, Wells A, Wilson MH, Servadei F (2019). Consensus statement from the international consensus meeting on the role of decompressive Craniectomy in the Management of Traumatic Brain Injury : consensus statement. Acta Neurochir.

[58] Regional Committees for Medical and Health Research Ethics (REC). Examples of avtivities that do not require approval from REC. Available from: https://helseforskning.etikkom.no/reglerogrutiner/soknadsplikt/sokerikkerek?p_dim=34999&_ikbLanguageCode=us. Accessed 6 July 2018.

